# Advances in Thermoelectric Materials Research

**DOI:** 10.1002/smsc.202500029

**Published:** 2025-02-26

**Authors:** Tiejun Zhu

**Affiliations:** ^1^ State Key Laboratory of Silicon and Advanced Semiconductor Materials School of Materials Science and Engineering Zhejiang University Hangzhou 310058 China

Thermoelectric materials can convert heat into electricity, and vice versa, offering a solution to versatile applications, including but not limited to power generation, solid‐state cooling, and precise temperature control. As an application‐driven research field, the rapid development of new technologies, such as artificial intelligence, the Internet of Things, flexible electronics, and deep space detection, has continuously renewed the demand for high‐efficiency thermoelectric materials and devices.

In the aspect of practical applications, no single thermoelectric material/device can satisfy all the application requirements in versatile scenarios, which means one should develop and design compatible thermoelectric materials and devices for specific applications. Meanwhile, in thermoelectric materials, the enhancement of the dimensionless figure of merit *zT* is always a core theme so that the assembled devices can perform at high efficiency. The past decade has witnessed the development of high‐performance thermoelectric materials that can work efficiently in different temperatures and the booming research on application‐driven thermoelectric devices.

In this context, we hope the special issue on “Advances in Thermoelectric Materials Research”, organized in *Small Science* is a timely contribution to the community. This special issue showcases 6 research articles and 4 review articles, presenting recent advances and perspectives on new thermoelectric materials, the relationship between microstructure and thermoelectric properties, and typical technological applications. This special issue has garnered attention from thermoelectric researchers in esteemed institutions worldwide, including Tsing University, Zhejiang University, Illinois Institute of Technology, Harbin Institute of Technology (Shenzhen), RWTH Aachen University, German Aerospace Center, University of Notre Dame, Queen Mary University of London, Queensland University of Technology, and Technical University of Darmstadt.

Bismuth telluride (Bi_2_Te_3_) alloys are currently the best thermoelectric materials near room temperature, which dominate the commercial thermoelectric markets. The further improvement of the thermoelectric performance and mechanical properties of Bi_2_Te_3_‐based alloys is crucial to promote the development and applications of near‐room temperature thermoelectric technology. Li and co‐workers^[^ ^1^ ^]^ comprehensively reviewed how the nanocomposite strategy can serve as an effective approach to improving both the thermoelectric and mechanical properties of Bi_2_Te_3_‐based alloys. The introduction of nanocomposite could effectively enhance the scattering of phonons and reduce the lattice thermal conductivity, while band bending induced by metal‐semiconductor or semiconductor‐semiconductor contacts might selectively impede the transport of low‐energy carriers, which is beneficial for the augmented Seebeck coefficient. In the fabrication of both p‐type and n‐type polycrystalline Bi_2_Te_3_‐based alloys, the donor‐like effect, describing the phenomenon of the increased electron density, often appears, which can significantly alter the thermoelectric performance. Zhu and his team^[^ ^2^ ^]^ found that the donor‐like effect in Bi_2_Te_3_‐based polycrystals originates from the oxygen‐adsorption‐induced evolution of the point defects. By strictly avoiding the adsorption to oxygen, the donor‐like effect can be eliminated, benefiting the reproducible fabrication of high‐performance Bi_2_Te_3_‐based polycrystalline materials.

Besides bismuth telluride, Wang and colleague^[^ ^3^ ^]^ successfully synthesized high‐quality tellurene‐like nanosheets and assembled nanostructured bulk materials using hot pressing. They achieved effective doping in the tellurene‐like materials with surface doping with chalcogenidometalates. The room temperature *zT* of the bulk sample based on tellurene‐like nanosheet is comparable to bulk Te. As a system characterized by the “phonon‐glass electron‐crystal”, Zintl phase compounds have attracted increasing research attention as promising thermoelectric materials. Zhang's team^[^ ^4^ ^]^ reported an ultralow lattice thermal conductivity of about 0.59 W m^−1^ K^−1^ at 300 K in CaAgSb, which is even lower than other Zintl phases. By combining the theoretical phonon calculations and atomic‐scale scanning transmission electron microscopy observations, they found the avoided crossing and flat phonon band effects, as well as the abundant structural defects, which are responsible for the low thermal conductivity. In another study, Yu and his colleagues^[^ ^5^ ^]^ found that the dopants, generally used to tune the electrical properties of thermoelectric semiconductors, can accumulate at grain boundaries. The segregation of Cu in the grain boundaries of PbSe material can reduce the potential barrier height of grain boundaries and thus weaken the effect of grain boundaries on charge carrier transport. Environmental‐friendly and cost‐effective Mg_2_(Si,Sn) thermoelectric material has also caught the attention of this special issue. Boor and co‐workers^[^ ^6^ ^]^ reported the diffusion of loosely bound Mg from the bulk towards the surface and subsequent oxidation result in the degraded thermoelectric performance of Mg_2_(Si,Sn). The understanding of the instability mechanism is important for the practical application of thermoelectric materials. In addition to the typical inorganic semiconductors, a review article on the design of intrinsically conductive metal‐organic frameworks for thermoelectric materials is provided by Nielsen and co‐workers.^[^ ^7^ ^]^

This special issue also presents a review article on the high‐throughput discovery of new thermoelectric materials by Zhang and his team.^[^ ^8^ ^]^ The integration of high‐throughput material processing and characterization techniques can efficiently speed up the exploration of new materials with potentially high thermoelectric performance. Additionally, the development of advanced manufacturing methods will facilitate the scalable assembly of thermoelectric devices. In another review article by Chen and colleagues,^[^ ^9^ ^]^ advances in magnetron sputtering‐fabricated flexible thermoelectric materials and devices are comprehensively discussed. As one of the most widely used thin‐film fabrication techniques, further addressing the current challenges of magnetron‐sputtering‐based deposition techniques will guide the development of highly efficient inorganic thermoelectric materials and devices. Beyond energy harvesting and solid‐state cooling, Xie and coworkers^[^ ^10^ ^]^ reported the study of intelligent sensing using thermoelectric materials, which represents a new research direction in the field.

As entering the conclusion of this special issue, we would like to express our cordial gratitude to all the contributors for their excellent research and insightful perspectives. We believe the broad coverage of this special issue, including new materials, microstructure and transport properties, theoretical calculations, and device fabrications, could inspire researchers toward advances in thermoelectric research. Last but not least, we would also like to acknowledge Dr. Ekaterina(Kate) Perets, the Editor‐in‐Chief of *Small Science*, and Dr. Jiaqi Li, for their help and leadership in making this special issue a success. Finally, we thank the reviewers for their constructive comments and suggestions to help maintain the high quality of articles in this special issue.

##  

## Conflict of Interest

The author declares no conflict of interest.
